# Draft Genome Sequence of Escherichia coli Strain UMD142

**DOI:** 10.1128/MRA.01009-20

**Published:** 2020-11-05

**Authors:** Tracy H. Hazen, Husain Poonawala, Kapil K. Saharia, Michael S. Donnenberg, David A. Rasko

**Affiliations:** a Institute for Genome Sciences, University of Maryland School of Medicine, Baltimore, Maryland, USA; b Department of Microbiology and Immunology, University of Maryland School of Medicine, Baltimore, Maryland, USA; c Department of Internal Medicine, Division of Infectious Diseases, Virginia Commonwealth University School of Medicine, Richmond, Virginia, USA; d Division of Infectious Diseases, Department of Medicine, University of Maryland Medical Center, Baltimore, Maryland, USA; e Institute of Human Virology, University of Maryland School of Medicine, Baltimore, Maryland, USA; Loyola University Chicago

## Abstract

Escherichia coli can be a harmless commensal organism or cause a range of diseases in humans, including diarrhea, urinary tract infections, meningitis, sepsis, and skin and soft tissue infections. Here, we describe the genome of an isolate that was associated with necrotizing fasciitis and the decompensation of previously undiagnosed cirrhosis.

## ANNOUNCEMENT

Escherichia coli has been linked with soft tissue infections, which can lead to secondary infections (1). The E. coli UMD142 isolate described in this submission was from a 48-year-old man with a history of morbid obesity, hypertension, gastrointestinal bleeding, diverticulitis, and chronic venous ulcer following deep vein thrombosis who presented to a community hospital with pain, swelling, and blisters of his leg. He was found to have features of septic shock, was admitted to the intensive care unit, and received empirical antibiotic therapy, vasopressors, and stress-dose steroids. He suffered acute kidney and liver decompensation and was transferred to the University of Maryland Medical Center, where deep debridement of necrotic tissue was performed. Further evaluation revealed cirrhosis, which ultimately required a liver transplant.

A deep wound culture obtained during intraoperative debridement of his wound yielded E. coli by inoculation onto MacConkey agar plates (Remel) containing 1 μg/ml ceftazidime and incubation at 37°C for 24 to 48 h. Lactose-fermenting colonies were identified as E. coli using API 20E identification strips or Vitek II testing (bioMérieux). For genomic DNA isolation, the bacteria were grown overnight in lysogeny broth, and the purified genomic DNA was collected from 1 ml of overnight culture using the GenElute genomic DNA kit (Sigma-Aldrich). Sequencing was performed by the University of Maryland School of Medicine, Institute for Genome Sciences, Genomics Resource Center (http://www.igs.umaryland.edu/resources/grc), with standard operating procedures. Library preparation was conducted using a Kapa kit for 150-bp paired-end sequencing, and sequencing was performed with an Illumina MiSeq system. A total of 11,715,063 raw read pairs of 150 bp were generated. Raw sequencing reads were subsampled to ∼180× with fastq-sample (https://github.com/fplaza/fastq-sample) and filtered to remove contaminating phiX reads using BBDuk of the BBTools software suite (https://sourceforge.net/projects/bbmap). The raw reads were also filtered to remove contaminating Illumina adaptor sequences and quality trimmed using Trimmomatic v0.36 (2). The resulting filtered reads were then assembled using SPAdes v3.14.0 (3). The assemblies were filtered to contain only contigs longer than 500 bp with k-mer coverage of ≥5×. All software was used with default values. The genome consists of 137 contigs with an *N*_50_ value of 209,377 bp and a final sequencing coverage of 172.8×. The resulting genome size is 5,208,771 bp with a G+C content of 50.81%. The genome was annotated with PGAP v4.12 (4). E. coli UMD142 was notable for the absence (as determined by examination of the PGAP annotation) of virulence factors described in previous reports of E. coli-associated necrotizing soft tissue infection (1). Many of these absent virulence factors overlap other extraintestinal E. coli strains. This genome serves as the reference for E. coli isolates from deep wounds and necrotizing fasciitis, as well as potentially cases of decompensation of liver function in the presence of cirrhosis.

### Data availability.

All data have been released, and the accession numbers are as follows. The assembly of the genome is under GenBank accession number MBPZ00000000, and the raw reads have been submitted to the SRA under accession number SRR12396409.
